# DICER1 RNase IIIb domain mutations are infrequent in testicular germ cell tumours

**DOI:** 10.1186/1756-0500-5-569

**Published:** 2012-10-15

**Authors:** Carmela M de Boer, Ronak Eini, Ad M Gillis, Hans Stoop, Leendert HJ Looijenga, Stefan J White

**Affiliations:** 1 Center for Reproduction and Development, Monash Institute of Medical Research, Monash University, Clayton, Australia; 2 Department of Pathology, Erasmus MC, University Medical Center Rotterdam, Josephine Nefkens Institute, Rotterdam, The Netherlands

**Keywords:** miRNA, DICER1, Cancer, Testicular germ cell tumours, Mutation detection

## Abstract

**Background:**

Testicular Germ Cell Tumours (TGCT) are the most frequently occurring malignancy in males from 15–45 years of age. They are derived from germ cells unable to undergo physiological maturation, although the genetic basis for this is poorly understood. A recent report showed that mutations in the RNase IIIb domain of DICER1, a micro-RNA (miRNA) processing enzyme, are common in non-epithelial ovarian cancers. *DICER1* mutations were found in 60% of Sertoli-Leydig cell tumours, clustering in four codons encoding metal-binding sites. Additional analysis of 14 TGCT DNA samples identified one case that also contained a mutation at one of these sites.

**Findings:**

A number of previous studies have shown that *DICER1* mutations are found in <1% of most cancers. To provide a more accurate estimate of the frequency of such mutations in TGCTs, we have analysed 96 TGCT samples using high resolution melting curve analysis for sequence variants in these four codons. Although we did not detect any mutations in any of these sites, we did identify a novel mutation (c.1725 R>Q) within the RNase IIIb domain in one TGCT sample, which was predicted to disturb DICER1 function.

**Conclusion:**

Overall our findings suggest a mutation frequency in TGCTs of ~1%. We conclude therefore that hot-spot mutations, frequently seen in Sertoli-Leydig cell tumours, are not common in TGCTs.

## Findings

### Background

Testicular germ cell tumours (TGCTs), also referred to as type II TGCTs, are the most common cancer affecting adolescent and young men, and are typically diagnosed around the age of 30. TGCTs are classified based on a number of features, including clinical characteristics and histological markers [1]. TGCTs are derived from immature germ cells that did not mature during embryonic development [2,3], and can be subdivided into seminomas and non-seminomas. Seminomas consist of transformed germ cells that do not exhibit overt pluripotency, although expressing SOX17, OCT3/4 and NANOG [4,5]. In contrast, non-seminomas are transformed germ cells that have reactivated pluripotency, as evidenced by the expression of SOX2, OCT3/4 and NANOG [6], three main regulators of pluripotency.

Micro-RNAs (miRNAs) play key roles in regulating mRNA levels [7]. All miRNAs are derived from precursor sequences, which are processed by DICER1 to form double stranded RNA duplexes. These duplexes consist of a principle miRNA strand and the (imperfectly) complementary miRNA strand, referred to as miRNA* [8].

Germline mutations in *DICER1* have been identified in patients with pleuropulmonary blastoma [9], often associated with goiter and Sertoli-Leydig cell tumours. A recent report described the identification of recurrent, somatic mutations in the *DICER1* gene in nonepithelial ovarian cancers [10]. The highest frequency of *DICER1* mutations were found in Sertoli-Leydig cell tumours, where 26 of 43 (60%) contained a somatic variant within one of four hot-spot codons. All four codons encode for acidic amino acids acting as metal binding sites within the RNase IIIb domain of DICER1. Mutations affecting any of these residues resulted in reduced RNase IIIb activity.

Additional analysis of other tumour types identified a somatic *DICER1* hotspot mutation in one of 14 TGCT samples, raising the possibility of mutations within this domain of DICER1 also playing a role in TGCT development. To better estimate the frequency of somatic variants within these regions in TGCTs, we have analysed 96 TGCT samples using High Resolution Melting Curve analysis, a powerful method widely used for identifying variants in genomic DNA [11,12].

### Results

**Figure 1:**
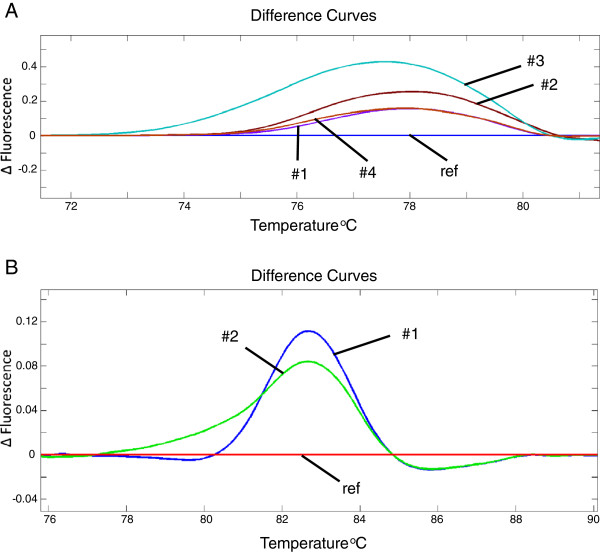
**HRM analysis detection of previously identified mutations within the DICER1 RNase IIIb domain.** Nucleotide and amino acid numbering are based on DICER1 reference sequence [NCBI:NM_177438]. 1**A**. Aberrant HRM curves resulting from four different heterozygous mutations, affecting amino acids 1705 and 1709 of DICER1. #1 = c.5113G>A (p.E1705K); #2 = c.5125G>A (p.D1709N); #3 = c.5126A>G(p.D1709G); #4 = c.5127T>A (p.D1709E); Ref = reference sequence. 1**B**. Aberrant HRM curves resulting from two different heterozygous mutations, affecting amino acids 1810 and 1813 of DICER1. #1 = c.5428G>T (p.D1810Y); #2 = c.5437G>C (E1813Q); Ref = reference sequence.

We have used HRM analysis to screen 96 TGCT samples for sequence variants in the four mutation hot-spots codons identified in the RNase IIIb domain of *DICER1*. To first demonstrate that HRM was able to detect the specific variants previously identified in *DICER1*, we created six different DNA templates (Table 1), each containing one of the *DICER1* mutations described in [10] that was identified in more than one sample. Combined, these six mutations cover 79% (26/33) of all cases where a mutation within one of the hot-spot codons was identified. For each variant, a heterozygous mutation was simulated by combining the variant template with an equimolar amount of a DNA template containing the reference sequence. As shown in Figure 1, all six variants could clearly be identified using HRM analysis.

**Table 1:** DNA constructs created to simulate DICER1 hot-spot mutations

Variant*	**Oligonucleotide sequences^**
1705_1709	
Reference sequence	5^′^-GGTGCTTGGTTATGAGGTAGTCCaaaatcgcatctcccaggaattctaagCGCTGGTAACAATCTGAGGG-3^′^
	3^′^-CCACGAACCAATACTCCATCAGGttttagcgtagagggtccttaagattgGCGACCATTGTTAGACTCCC-5^′^
c.5113G>A p.E1705K	5^′^-GGTGCTTGGTTATGAGGTAGTCCaaaatcgcatctcccaggaatt**T**taagCGCTGGTAACAATCTGAGGG-3^′^
	3^′^-CCACGAACCAATACTCCATCAGGttttagcgtagagggtccttaa**A**attgGCGACCATTGTTAGACTCCC-5^′^
c.5125G>A p.D1709N	5^′^-GGTGCTTGGTTATGAGGTAGTCCaaaatcgcat**T**tcccaggaattctaagCGCTGGTAACAATCTGAGGG-3^′^
	3^′^-CCACGAACCAATACTCCATCAGGttttagcgta**A**agggtccttaagattgGCGACCATTGTTAGACTCCC-5^′^
c.5126A>G p.D1709G	5^′^-GGTGCTTGGTTATGAGGTAGTCCaaaatcgca**C**ctcccaggaattctaagCGCTGGTAACAATCTGAGGG-3^′^
	3^′^-CCACGAACCAATACTCCATCAGGttttagcgt**G**gagggtccttaagattgGCGACCATTGTTAGACTCCC-5^′^
c.5127T>A p.D1709E	5^′^-GGTGCTTGGTTATGAGGTAGTCCaaaatcgc**T**tctcccaggaattctaagCGCTGGTAACAATCTGAGGG-3^′^
	3^′^-CCACGAACCAATACTCCATCAGGttttagcg**A**agagggtccttaagattgGCGACCATTGTTAGACTCCC-5^′^
1810_1813	
Reference sequence	5^′^-CATGTAAATGGCACCAGCAAgcgactcaaaaatatcccccatggCCTTTGGAACTTCAATATCCTCTT-3^′^
	3^′^-GTACATTTACCGTGGTCGTTcgctgagtttttatagggggtaccGGAAACCTTGAAGTTATAGGAGAA-5^′^
c.5428G>T p.D1810Y	5^′^-CATGTAAATGGCACCAGCAAgcgactcaaaaatat**A**ccccatggCCTTTGGAACTTCAATATCCTCTT-3^′^
	3^′^-GTACATTTACCGTGGTCGTTcgctgagtttttata**T**ggggtaccGGAAACCTTGAAGTTATAGGAGAA-5^′^
c.5437G>C p.E1813Q	5^′^-CATGTAAATGGCACCAGCAAgcgact**G**aaaaatatcccccatggCCTTTGGAACTTCAATATCCTCTT-3^′^
	3^′^-GTACATTTACCGTGGTCGTTcgctga**C**tttttatagggggtaccGGAAACCTTGAAGTTATAGGAGAA-5^′^

* Nucleotide and amino acid numbering are based on DICER1 reference sequence [GenBank:NM_177438].

^ Priming sequences for PCR amplification (using primers listed in Table 2) are in capital letters. The nucleotides representing the mutations are in bold.

We then used PCR primers (Table 2) to amplify the corresponding genomic regions in 96 TGCT samples to screen for these six variants. No samples showed an aberrant melting curve.

**Table 2:** PCR amplification primers used in this study

Genomic region covered*	**Forward primer (5′-3′)**	**Reverse primer (5′-3′)**	**Product size**	**Comment^**
chr14:95560431-95560500	GGTGCTTGGTTATGAGGTAGTCC	CCCTCAGATTGTTACCAGCG	70 bp	Product includes codons 1705–1709 of DICER1; primer sequences from this study
chr14:95557604-95557671	CATGTAAATGGCACCAGCAA	AAGAGGATATTGAAGTTCCAAAGG	68 bp	Product includes codons 1810–1813 of DICER1; primer sequences from this study
chr14:95560345-95560533	CTTCTGCACAAGCTTACGGTTCCA	CAGCGATGCAAAGATGGTGTTGT	188 bp	Product includes codons 1705–1709 of DICER1; primer sequences from [10].
chr14:95557565-95557759	TGGACTGCCTGTAAAAGTGG	ACACACCTGCCAGACTGTCTCC	194 bp	Product includes codons 1810–1813 of DICER1; primer sequences from [10].

* Genomic location is based on reference sequence hg19.

^ Amino acid numbering is based on DICER1 reference sequence [GenBank:NM_177438].

**Figure 2:**
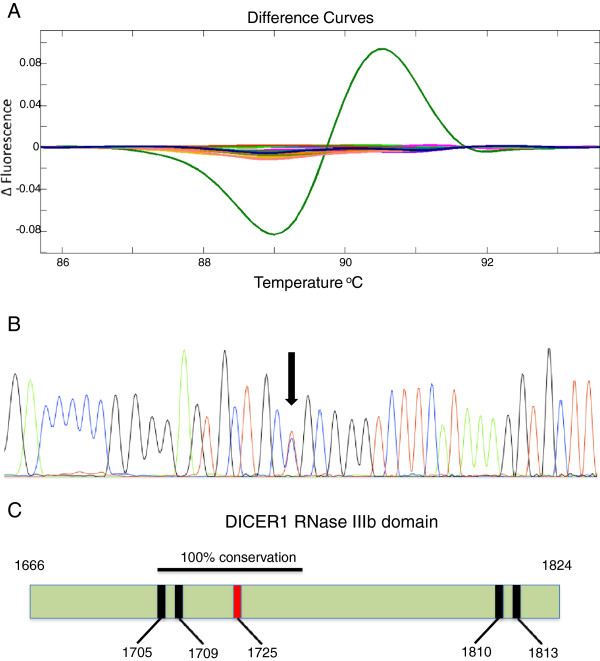
**A novel mutation identified within the DICER1 RNase IIIb domain in a single seminoma sample.** Amino acid numbering is based on DICER1 reference sequence [GenBank:NM_177438]. 2**A**. The aberrant HRM curve corresponding to the *DICER1* mutation. The curves on the baseline represent samples without a sequence variant (this was confirmed with Sanger sequencing in one sample). 2**B**. A Sanger sequencing trace from the sample that showed the aberrant HRM curve in 2A. The heterozygous sequence variant is indicated by an arrow. This mutation is predicted to change an Arginine to a Glutamine at position 1725 of DICER1. 2**C**. The RNase IIIb domain in DICER1, containing amino acids 1666–1824. The location of the mutation (R1725Q) identified in this study is shown by the red bar. The locations of the four metal-binding residues (all acidic amino acids) frequently mutated in Sertoli-Leydig cell tumours are shown by black bars. The region of 100% conservation across at least 42 species at the amino acid level (residues 1705–1741) is indicated by the horizontal black bar.

As this initial analysis only covered a small amount of genomic sequence (70 bp and 68 bp for the 1705/1709 and 1810/1813 codons respectively), we rescreened the same 96 TGCT samples using primers described in [10] (Table 2). These reactions generated products of 188 bp and 194 bp, covering more of the RNase IIIb domain. In these expanded assays, only one sample (a seminoma) showed an aberrant curve with either primer pair (Figure 2A). Sanger sequencing revealed a G>A transition (Figure 2B), predicted to change an Arginine to a Glutamine at position 1725 (Figure 2C). This variant is not listed in the 1000 Genome Project data [13], nor is it present in Catalogue of Somatic Mutations in Cancer (COSMIC), a database that curates mutations from a range of different cancers [14].

Although the affected amino acid is not acidic, and not predicted to directly function as a metal-binding site, it is within a contiguous sequence of 37 amino acids that show 100% conservation across at least 42 species. This supports the hypothesis that this region within the RNase IIIb domain is critical for normal *DICER1* function. Indeed, analysis using PolyPhen2 [15] predicts the impact of this variant to be “probably damaging” (score 1.0, sensitivity 0.0, specificity 1.0).

### Discussion

Somatic sequence variants are rare in TGCTs. Analysis of 518 kinase genes in seven seminoma and six non-seminoma samples identified a single somatic point mutation, with an estimated mutation frequency of 0.12 per Mb [16]. A small number of genes are recurrently mutated in TGCT, including *KIT*, *KRAS2* and *BRAF*[17,18], but in each case these represent <10% of tumours analysed.

Although Heravi-Moussavi *et al*. found *DICER1* mutations in non-epithelial tumours, including a TGCT, >85% of these mutations were restricted to Sertoli-Leydig cell tumours of the ovary. Sertoli-Leydig tumours are composed of both Sertoli and Leydig cells, which are cell types normally found in the testis [19]. They are derived from the sex cords, which originate from the gonadal ridge prior to sex determination. In contrast, seminomas and non-seminomas are derived from germ cells that have not undergone appropriate maturation. As such, these two tumour types represent distinct cell lineages.

The mutations in the RNaseIIIb domain were shown to alter rather than abolish DICER1 activity, and it was proposed that a specific miRNA expression profile would be derived as a result of changes in RNase IIIb activity [10]. The RNase IIIb domain is known to cut the miRNA strand, with the miRNA* strand being cut by the RNase IIIa domain [20]. Impaired RNase IIIb activity would therefore be expected to result in a relative increase in miRNA* production.

Several studies have shown increased expression of certain miRNAs in TGCTs, primarily the miR302 and miR37-373 clusters [21,22]. Indeed, expression of these miRNAs is a hallmark of these cancers. These miRNAs are also upregulated in embryonic stem cells and other pluripotent cell types [23], and their high expression levels in TGCTs are thought to represent the pluripotent cell type of origin rather than specific genetic mutations affecting expression levels [24]. We have recently performed copy number variation and sequence analysis of these miRNA loci in a large cohort of TGCTs, and did not identify any mutations likely to be responsible for altering miRNA levels (unpublished observations, de Boer *et al*.).

### Conclusion

In conclusion, we show that previously described hot-spot mutations within the RNase IIIb domain of DICER1 are not frequent in TGCTs. It is likely that the high rate of recurrent mutations observed in Sertoli-Leydig cell tumours is due to specific characteristics of this tumour type.

### Materials and methods

#### TGCT samples

All TGCT DNA samples were extracted at Erasmus University Medical Center, Rotterdam, the Netherlands. The 96 samples consisted of 32 primary seminomas and 64 primary non-seminomas. None had been treated by either chemotherapy or irradiation. Use of tissue samples for scientific reasons was approved by an institutional review board (MEC 02.981 and CCR2041). Samples were used according to the “Code for Proper Secondary Use of Human Tissue in The Netherlands” as developed by the Dutch Federation of Medical Scientific Societies (FMWV) Version 2002, update 2011). Genomic DNA was isolated from peripheral blood lymphocytes following standard protocols.

This project was approved by the Monash University Human Research Ethics Committee, #CF11/1841.

#### Mutation analysis

All oligonucleotides were ordered from Sigma-Aldrich (Castle Hill, Australia). Double-stranded DNA sequences containing either the reference sequence, or one of the six most commonly identified variants in [10], were generated by annealing two complementary single-stranded oligonucleotides of the appropriate sequences (Table 1).

Primers for PCR amplification are listed in Table 2. Amplification reactions were performed in 10 μl reaction volumes, consisting of HRM Master Mix (Idaho Technologies, USA), 5 μM each of forward and reverse primer, and either 25 ng genomic DNA (TGCT samples) or 1 fmol of variant DNA template.

PCR reactions were carried out under the following conditions; an initial hold at 95°C for 2 min, followed by 45 cycles of 94°C for 30 sec and 58°C for 30 sec. PCR products were analysed in a 96 well plate in the LightScanner (Idaho Technologies, USA). The HRM settings for the LightScanner were as follows; start temperature of 70°C, end temperature at 96°C, with a hold temperature at 67°C. HRM curves were normalized using GeneMelt software supplied with the instrument, and samples that showed an aberrant melting curve were analysed by Sanger sequencing at the Gandel Charitable Trust Sequencing Centre at the Monash Health Translation Precinct, Melbourne, Australia. Sequence traces were visually assessed using 4Peaks software (Mek&Tosj, Amsterdam, the Netherlands).

#### Bioinformatic analysis

The predicted effect of missense mutations was determined using PolyPhen2 [15].

## Competing interests

The authors declare that they have no competing interests.

## Authors’ contributions

SdB performed the genetic studies and helped draft the manuscript. RE, AG, and HS performed the molecular analysis and preparation of the samples. LL conceived of the study, participated in its design and coordination and helped draft the manuscript. SW conceived of the study, participated in its design and coordination and drafted the manuscript. All authors read and approved the final manuscript.
